# Interactions with conspecific outsiders as drivers of cognitive evolution

**DOI:** 10.1038/s41467-020-18780-3

**Published:** 2020-10-06

**Authors:** Benjamin J. Ashton, Patrick Kennedy, Andrew N. Radford

**Affiliations:** 1 School of Biological Sciences, University of Bristol, 24 Tyndall Avenue, Bristol, BS8 1TQ UK; 2 Department of Biological Sciences, Macquarie University, Sydney, NSW 2109 Australia

**Keywords:** Behavioural ecology, Social evolution, Social behaviour, Animal behaviour

## Abstract

The social intelligence hypothesis (SIH) posits that within-group interactions drive cognitive evolution, but it has received equivocal support. We argue the SIH overlooks a major component of social life: interactions with conspecific outsiders. Competition for vital resources means conspecific outsiders present myriad threats and opportunities in all animal taxa across the social spectrum (from individuals to groups). We detail cognitive challenges generated by conspecific outsiders, arguing these select for ‘Napoleonic’ intelligence; explain potential influences on the SIH; and highlight important considerations when empirically testing these ideas. Including interactions with conspecific outsiders may substantially improve our understanding of cognitive evolution.

## Abstract (web summary)

The social intelligence hypothesis predicts that social organisms tend to be more intelligent because within-group interactions drive cognitive evolution. Here, authors propose that conspecific outsiders can be just as important in selecting for sophisticated cognitive adaptations.

## Introduction

Cognitive evolution is one of the most hotly debated topics in biology, with considerable uncertainty remaining about the likely drivers. Some of the earliest hypotheses for the evolution of intelligence in non-human animals focused on ecological factors, such as the cognitive demands associated with locating spatially and temporally unpredictable food sources^1^. Several comparative studies have supported the predictions of the so-called ecological intelligence hypothesis; for instance, environmental variation and diet have been identified as key selection pressures for brain-size evolution in birds and primates, respectively^2–4^. However, a major body of conceptual and empirical work indicates that the social environment can also play a crucial role in cognitive evolution^5–7^.

To date, predictions about the social drivers of cognitive evolution have largely focused on within-group interactions^8^. Byrne and Whiten^5^ hypothesised that competitive interactions among groupmates (e.g., for food and/or mates) select for cognitively demanding behaviours, such as tactical deception, social manipulation and political manoeuvring. Conversely, Dunbar^6^ argued that the cooperative aspects of group living, including the need to create functional, cohesive, bonded groups to solve ecological problems, select for greater cognition. Positive actions between group members, such as the trading of commodities (e.g., grooming for coalitionary support) and the contingent rewarding of cooperative acts, may also generate cognitive demands^9–11^. Collectively, these within-group aspects of social life are argued to drive cognitive evolution^12,13^ and form the basis of the social intelligence hypothesis (SIH) (see Dunbar and Shultz^14^ for a review of the SIH and its variants). The SIH was initially conceived with primates in mind and there is strong evidence to suggest that the social environment explains some of the cognitive variation observed in this taxonomic group (e.g., a positive relationship between group size and brain size)^15^. However, some primate studies have not found this relationship^2,4^ and it is also not so apparent in other taxa; pair-bondedness (indicative of strong social relationships) is associated with large brains in some avian^16^ and non-primate mammalian^17^ species, but there are also a number of studies reporting no relationship between sociality and cognition in these taxa^3^. The SIH, as currently framed, therefore receives somewhat equivocal support and leaves a significant amount of cognitive variation unexplained.

We argue that considerable cognitive variation may have remained unexplained because the SIH, and empirical tests of it, overlook a second major axis of social variation in animals: interactions with conspecific outsiders. From solitary species to those living in complex groups, in all animal taxa from invertebrates to primates, interactions with conspecific outsiders are commonplace^18–20^ and their profound effects on sociality have become increasingly recognised^21–24^. Rival individuals, pairs or groups may compete for valuable resources such as mating opportunities, breeding positions, food or territories^25–27^. Such competition and associated threats and opportunities likely create selection pressures for cognitive traits that aid success. The idea that conspecific outsiders could influence cognitive evolution has been proposed before with respect to human evolution^28,29^. However, discussion of the cognitive consequences of outsider threats in non-human animals has largely focused on the challenges posed by predators^15^; the possible influence of conspecific outsiders on cognitive evolution has been largely ignored and untested (for notable exceptions, see refs. ^30,31^). Conspecific outsiders differ in several important ways from predators. For example, although predators represent only a threat, conspecific outsiders can present threats and opportunities. In addition, some threats posed by outsiders, such as those to mating or breeding positions, are unique to conspecifics. Finally, the roles of ‘predator’ and ‘prey’ are split between different species, whereas the roles of both parties (i.e., threatener and threatened) are played by members of the same species in interactions with conspecific outsiders. Although interactions with heterospecifics—both antagonistic ones (e.g., with predators) and those of a cooperative nature (e.g., between cleanerfish and clients)—undoubtedly pose cognitive challenges and are worthy of study, we focus here on conspecific outsiders. We term the ability to exploit interactions with conspecific outsiders to an actor’s advantage as ‘Napoleonic’ intelligence, given the strategic intelligence synonymous with Napoleon Bonaparte.

**Fig. 1:**
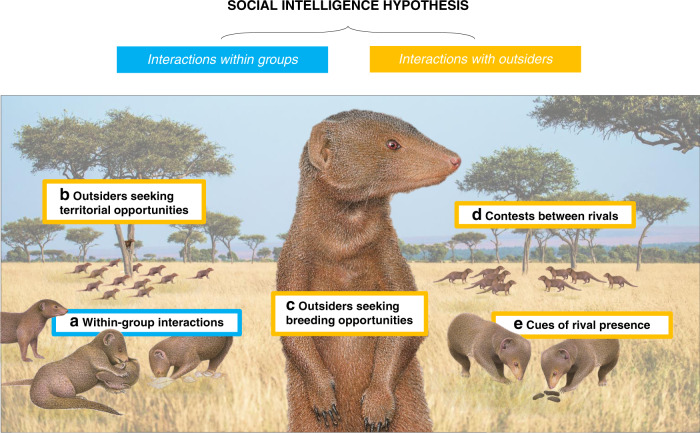
Two aspects of the social intelligence hypothesis (SIH). Within-group social intelligence is the capacity to succeed in within-group interactions (**a**); it represents the well-established basis of the SIH as currently framed^5, 6^. We argue that the capacity to succeed in interactions with conspecific outsiders should be included in the SIH for a more complete representation of the social environment. Significant cognitive challenges with respect to outsider threats and opportunities include those arising from **b** conflict with rivals over resources and territory space, **c** conflict with rivals over breeding or mating opportunities, **d** contest dynamics when there are adversarial interactions between rivals and **e** the evaluation of secondary cues (e.g., faecal deposits) containing information about conspecifics. NB: here we use the dwarf mongoose (*Helogale parvula*), a cooperatively breeding species, to illustrate some of the cognitive challenges posed by outsiders. However, such challenges and resulting Napoleonic intelligence are relevant across the social spectrum (including solitary and pair-bonded species, and those living in a variety of group structures). Dwarf mongoose illustrations: Martin Aveling. Landscape: David Clode.

Here we offer a case for the full inclusion of conspecific outsider interactions and resulting Napoleonic intelligence in the SIH. We begin by detailing the cognitive challenges presented by conspecific outsiders (hereafter referred to as ‘outsiders’), considering both those relevant across the whole social spectrum and additional challenges pertinent to group-living species. We then explain the potential influences of outsider interactions on the SIH: we describe the different selection pressures exerted by groupmates and outsiders, indicate how these social axes can interact additively or synergistically to influence cognition and assess intraspecific variation (e.g., between sexes) in selection pressures. Finally, we outline how the extension of the SIH to include outsider interactions can be tested empirically. Our argument is that both within-group and outsider interactions are crucial drivers of social intelligence (Fig. 1).

## Cognitive challenges arising from interactions with conspecific outsiders

Outside threats and opportunities present a variety of cognitively demanding challenges that likely generate consequences both plastically within the lifetime of individuals and on an evolutionary scale. Animal contests in general have been proposed to favour cognitive traits underpinning three broad skillsets—opponent evaluation, own status evaluation and assessment strategies^31^— all of which are applicable to interactions with outsiders. Individuals benefit from a capacity to assess rival characteristics and intent in relation to their own characteristics and to be able to appraise and respond to developments during encounters. More broadly, as individuals navigate the social context of competition with outsiders, there may be value in possessing abilities to change behaviour based on previous encounters, to remember rivals and spatial locations, to process varied, infrequent and unreliable information, and to make informed decisions. Cognitive traits relating to perception, learning and memory are therefore as applicable to interactions with outsiders as other conflict scenarios^32^. We consider in turn those cognitive challenges presented by outsiders that are relevant across the full social spectrum (from solitary and pair-bonded animals to those living in groups) and those cognitive challenges that are of particular relevance to group-living species.

### Cognitive challenges across the social spectrum

To maximise opportunities and to reduce the threats and costs associated with outsiders, decisions are needed about whether and how to engage with rivals. Decision-making regarding interactions with outsiders is often influenced by their identity. For example, opportunities to obtain breeding positions or extra-pair/extra-group matings vary between neighbouring territories^25,33^. Similarly, from a threat perspective, it may be adaptive to avoid rivals of greater resource-holding potential (RHP); relative RHP, and therefore the odds of winning a contest, can depend on the size of individuals, the strength of a pair bond or the size and composition of groups^27,34,35^. Furthermore, the decision to engage in territorial behaviour may require an ability to infer relatedness to specific groups^36^ (and therefore indirect fitness consequences of outsider interactions). In general, opponent recognition can arise from such processes as habituation learning^37^, categorisation of different classes and even individual recognition^38^. Deciding whether to engage in contests with conspecific outsiders is made all the more important given the inherent risks: although the potential benefits (e.g., in terms of resource acquisition) are high, the costs can be substantial as there may be escalation to violence and possible injury or death^25,39^. Even when the costs are not this extreme, engagement takes time and energy, and can have consequences for the harmony of pairs or groups^21,40^. During outsider interactions, strategic behaviour may be modified in relation to different third-party audiences^41^ (both insiders and outsiders). Acquisition, retention and assessment of relevant information helps individuals make complex decisions about, for instance, whether to invest in resource defence, attack a territorial neighbour or attempt dispersal or sneaky matings, and how to react during contests.

Enhanced spatial memory is also likely to be important in the context of outsider conflict and opportunities (e.g., remembering the position of territory boundaries and the locations of previous interactions). For example, the use of cognitive maps can allow avoidance of areas where costly contests with rivals tend to occur^42^ or behavioural preparation for potential encounters when in zones of conflict^43^. Conversely, this information may also be used to identify opportunities for territory expansion. In many species, neighbours and non-neighbours (‘strangers’) present different threat levels; in some cases, strangers are more threatening (contexts in which neighbours are known as ‘dear enemies’), whereas sometimes neighbours present the greater threat (contexts in which neighbours are known as ‘nasty neighbours’)^44,45^. Knowledge about the location of specific neighbouring territories is important to ensure a suitable response to neighbours vs. strangers, but also because, in dear-enemy situations, stronger responses are favoured towards neighbours on the ‘wrong’ territorial boundary compared to where they are expected spatially^46^. Selection should favour a capacity to obtain, update and retain this spatial information.

To add to the cognitive challenge of monitoring and assessing outside threats and opportunities, relationships are dynamic and information about outsiders may only be available infrequently and often just from secondary cues. In territorial species, mortality, take-overs and dispersal mean that the identity of neighbours will change across time. The relationship with a given neighbour can also alter with repeated interactions^44^. For instance, a recent incomer might initially be viewed as a nasty neighbour but could become a dear enemy as knowledge and familiarity increase; responses can also change dynamically if outsiders intrude more frequently or become more aggressive^47,48^. More generally, and equally relevant to non-territorial species, threats and opportunities can fluctuate with context^44^; mating opportunities, e.g., will only be available when there are fertile females in the population^26^. On a spatial scale, territorial boundaries can change, as owners expand or contract the area over which they control access^19^. Compounding the cognitive challenge of tracking and reassessing such situations, information regarding outsiders might be received relatively infrequently; encounters with rivals or indicators of their presence may occur only every few days or weeks^49^. Relevant information can be obtained from deliberate exchanges between rivals^34,50^ or from secondary cues (e.g., faecal deposits^51,52^). The cognitive demands of signal detection, deception, interpretation and response, as well as integrating information of differing reliability from direct and indirect sources, is likely considerable^53^.

### Additional cognitive challenges arising in group-living species

Group-living species encompass a range of social structures, from territorial, cooperatively breeding animals where a ‘group’ is easily defined to fission–fusion societies (where the ‘group’ is more ambiguous and could refer to the ‘parent group’ or ‘sub-groups’) and societies where ‘group’ identity varies with context. However, all can experience additional, and qualitatively different, cognitive challenges to those arising in species with simpler social structures. For instance, in the context of dynamic outsider characteristics, there can be changes in the identity of a whole neighbouring group, a subset of the group or just a single member. This alters both the collective threat and the potential threats and opportunities presented to individuals (e.g., from individual roving males seeking breeding opportunities^54^). Furthermore, information about multiple group members, available from individually distinct scent marks and calls^55,56^, requires more elaborate cognitive processing than that from single outsiders. Additional inferential challenges arise when others shift between ‘insider’ and ‘outsider’ status depending on social context (e.g., foraging groups vs. breeding groups).

Direct contests between groups may generate cognitively demanding dynamics. When groups fight, two main strategies are possible: members of a larger group could concentrate attacks on single-rival individuals or individuals from opposing groups could compete in one-on-one battles. Lanchester^57^ proposed that the former is governed by a ‘square law’, which predicts that numerical superiority leads to victory, whereas the latter is governed by a ‘linear law’ such that the fighting ability of individuals is the more important factor. Although initially conceived with humans in mind, Lanchester’s laws have been found to operate across a wide range of taxa, from primates^58^ to insects^59^. Thus, assessment of the size and ability of rival forces is likely crucial when deciding how to engage with them in contests, both initially and if a change in strategy is required during an interaction. As relative group size is often a strong determiner of between-group contest outcomes^60^, selection may favour a capacity to assess numerical differences (‘numerosity’) between own and rival groups^50^. In general, learning will be important in developing contest skills, including signalling components, fighting tactics or the ability to adjust behaviour in response to ongoing dynamics^31^.

Outsider threats can also affect within-group interactions. The motivations of individual actors with respect to between-group interactions have been repeatedly shown to differ substantially depending on, e.g., their age, sex, reproductive strategy, body condition and dominance status^60–63^. For instance, females often engage in between-group contests more frequently than males when the dispute is over resources such as territory or food^62^, whereas males of many species engage in between-group contests more readily when disputes are over access to females^62,64^. Similarly, dominant and subordinate group members may have more or less to gain from defence against different outsiders^60,65^. Resolving such conflicts of interest can foster the emergence of within-group punishment and deterrence either during or after between-group interactions^21,61^. Conversely, when the interests of individual group members are aligned, individuals participating more in contests with rivals may receive rewards, such as increased affiliative behaviour^21,66,67^. The tracking of individual contributions and the decision-making processes leading to resultant within-group agonistic and affiliative behaviours likely requires considerable investment in memory and accounting about the status of different relationships. Furthermore, monitoring whether these behaviours have any effect on subsequent contributions to interactions with outsiders^66^, especially interactions that occur relatively infrequently, introduces inferential challenges associated with between-group conflict.

## Integration of outsider interactions in the SIH

**Fig. 2:**
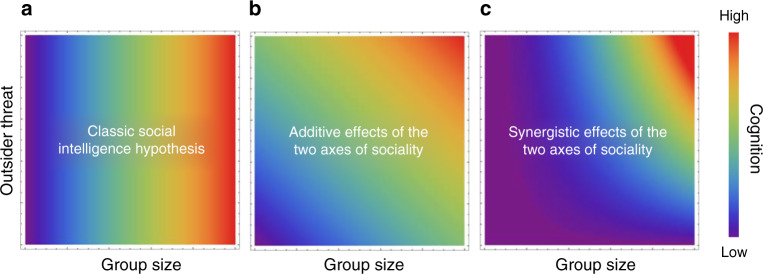
Potential effects of conspecific outsider threat on cognition. **a** The classic social intelligence hypothesis focuses only on one axis of sociality (within-group interactions, often using group size as a proxy) in driving cognitive evolution; variation in conspecific outsider threats is ignored. Combining within-group and outsider interactions could produce **b** an additive or **c** a synergistic effect on cognitive evolution.

A clear relationship between sociality and cognition, the main prediction of the SIH (Fig. 2a), has not consistently emerged^2,3,8,12^ and studies often report much unexplained variation. For instance, species with similar group sizes often show large variation in brain size that is not explicable when focusing solely on social intelligence associated with interactions within groups^2,4^. Existing proposed explanations for this brain-size variation include (1) differences in non-social ecological factors^2^, (2) physical constraints on brain size^12^ and (3) resource constraints on energetically expensive brains^12^. However, as the extent and nature of outsider interactions varies considerably between species, we argue that there are several reasons why including this social axis (and resulting Napoleonic intelligence) may improve the predictive power of the SIH.

Outsider interactions can select for higher cognitive abilities in both similar and different ways to within-group interactions. On the one hand, both require optimal responses to the behaviour (or predicted behaviour) of others. Here, the two axes may combine to promote higher cognition through selection on the same specific domain. On the other hand, outsider interactions often differ qualitatively from within-group interactions (Table 1). For instance, conflict within groups may favour a capacity for transitive reasoning in dominance hierarchies (based on individual recognition)^31,68^, which is likely often to be of limited use with respect to outsiders (but see ref. ^69^ for transitive inference between territory-holders). By contrast, conflict with outsiders may favour long-term spatial memory, which is likely to be of limited use in terms of within-group conflict. In such cases, the two social axes may promote different domain-specific cognitive abilities and the combined pressure on multiple domains may contribute to enhanced selection on domain-general intelligence.

**Table 1:** Key potential differences between selective pressures on cognitive evolution arising as a result of interactions within groups and with conspecific outsiders.

Within groups	With conspecific outsiders
Only relevant to group-living species	Relevant across the social spectrum from solitary to group-living species
Often frequent interactions between actors	Often rare episodic interactions between actors
Little spatial component	Strong spatial component (e.g., spatial memory about volatile territorial borders and disputed resources)
Often visual, reliable information about interactions	Information often obtained from non-visual secondary sources (such as auditory cues or scent marks) with reduced reliability
Regularly updated information: actor is likely to know about changes in within-group ‘politics’ rapidly	Infrequent opportunities for information: actor’s information may lag behind events
Transitive inference is often important for negotiating within-group dominance hierarchies	Transitive inference is unlikely to be of general importance

The selective pressures imposed by the two axes of sociality might interact in different ways. An additive scenario would involve a step along each axis boosting cognition by a fixed amount regardless of the value on the other axis (Fig. 2b). Alternatively, a synergistic scenario would involve both axes combining to drive disproportionately high cognitive abilities in contexts where high levels of within-group conflict are matched by high levels of outsider conflict (Fig. 2c). Two forms of synergistic effects can be envisaged. First, synergy may be a result of the way domain-general intelligence arises within the brain. Exposure to both within-group and outsider interactions may increase the diversity of social tasks. Consequent selection on several cognitive domains in parallel may boost domain-general intelligence more than selection on only a limited number of cognitive tasks. A second form of synergy may arise when an increase along one axis of sociality pushes the organism further along the other axis. Indeed, outsider threats may amplify the value of within-group social intelligence: within-group conflict can arise, for instance, through access to outgroup mates^54^ or disagreements about the optimum investment in between-group aggression^70^. Such non-additive effects may reduce the chances of confirming the predictions of the SIH as currently framed: e.g., in a species with large groups and low outsider threat, the effects of group size on general intelligence (and, by implication, brain size) may be disproportionately lower than in an equivalent species with high outsider threat.

It is likely that the selection pressures exerted by the social environment on cognitive evolution will vary between individuals. For instance, threats from outsiders may generate sex-specific challenges that result in sexually dimorphic cognitive traits. Indeed, there is evidence that sexual selection drives sexual dimorphism in the brain^71^ and one element of outsider threat, extra-pair or extra-group paternity, has already been linked to sexually dimorphic brains in birds^72^. High levels of breeding threat might select for behaviours such as territorial defence, elaborate courtship and mate guarding; as these behaviours entail several cognitive challenges, larger brains could evolve in the threatened sex. Particular components of outsider threats may generate cognitive selection pressures on specific types or classes of individuals.

Finally, we suggest a form of intrigue that may arise with respect to outsider interactions in group-living species: cognitive free-riding, where individuals might exploit the cognitive investment of others. For instance, a need to invest substantial cognitive effort into spatial memory to recall and monitor territorial boundaries may generate a collective-action problem^73^. This is because cognition is individually costly^74^—investment in cognition may come at the expense of lifespan^75^ or investments in other fitness-boosting functions, including digestion^76^ and immunity^77^—and at least some of the benefits are realised by the group as a whole. Group members who ‘parasitise’ the cognitive investments of other group members may therefore enjoy a within-group advantage, freeing cognitive resources for self-interest. Parasitically outsourcing cognitive abilities is rational when they do not confer a significant within-group advantage to individuals (e.g., spatial memory; Table 1). Thus, investment in aspects of cognition may itself become a source of within-group conflict, driving the evolution of divergent cognitive strategies^78^ within species.

## Testing predictions

To test our proposed expansion of the SIH, including both major axes of sociality, we advocate careful consideration of the metrics used to assess outsider interactions and when they are applicable, and the use of a complementary suite of intraspecific and interspecific approaches.

### Outsider metrics

**Fig. 3:**
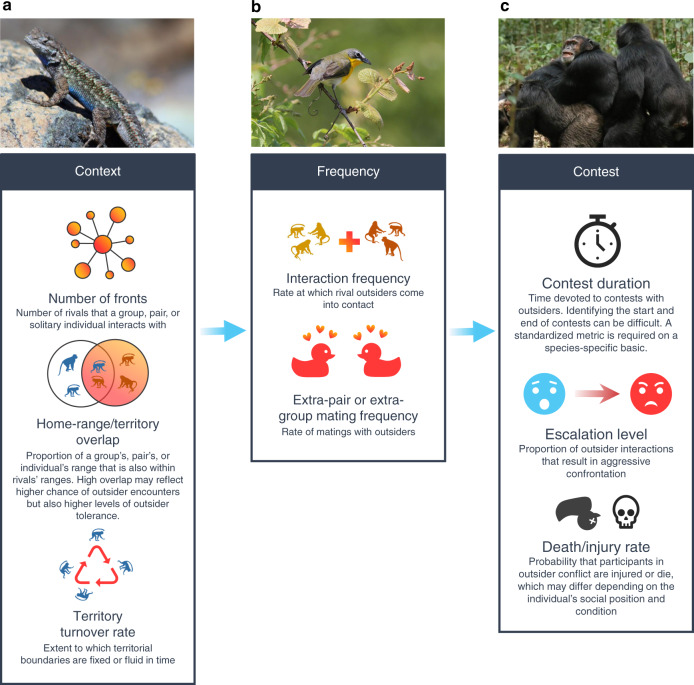
A plurality of metrics is required to measure interactions with conspecific outsiders at different scales. **a** ‘Context’ refers to the social landscape in which conspecific outsider interactions occur. In solitary western fence lizards (*Sceloporus occidentalis*), e.g., long-term observations reveal considerable home-range and territorial overlap^98^. Lizard photograph: D. A. Hofmann. **b** ‘Frequency’ refers to the rate of relevant interactions with conspecific outsiders. Many socially monogamous birds have high levels of extra-pair mating; for instance, in pair-bonded yellow-breasted chats (*Icteria virens*), telemetry reveals nocturnal visits to other territories for likely mating opportunities^99^. Yellow-breasted chat photograph: E. Willoughby. **c** ‘Contests’ refers to the characteristics of actual interactions between conspecific rivals. In chimpanzees (*Pan troglodytes*), e.g., violent between-group contests can have dramatic consequences for social evolution^100^. Chimpanzee photograph: L. Samuni.

Clear metrics of outsider threats and opportunities are needed. We suggest three main categories to capture the level of competition with outsiders, all of which are relevant to individuals, pairs and groups (Fig. 3); as with predation^79^, these range from measures of the overall risk level to those relating to specific events. First, quantification of the broad context—the landscape of outsider pressure (e.g., number of neighbours and territorial turnover rate). A high threat (or ‘shadow’) of conflict may actually cause neighbours to avoid escalation in their interactions but still play an important role in shaping social dynamics^80^ and, in turn, cognition. Second, quantification of the frequency of interactions within this contextual landscape, both in terms of the encounter rate between rivals (e.g., between-group interaction frequency) and the rate at which specific outcomes arise (e.g., extra-pair or extra-group paternity). Third, quantification of parameters relating to actual contests with outsiders (e.g., duration and level of escalation).

No single factor measures competition with outsiders in its entirety. One reason for this is that different types of threat, which are not necessarily strongly correlated, may be captured by different metrics. For instance, challenges to reproduction may best be indicated by extra-pair or extra-group paternity rates^72^, whereas threats to territory space may be better captured by the occurrence of agonistic encounters between rival individuals, pairs or groups^81,82^. Moreover, a single metric may not provide a clear-cut reflection of competition level across species. For example, one primate study of brain size has used greater home-range overlap to indicate greater between-group competition for resources^30^, but overlap could equally be indicative of higher tolerance in some species. Similarly, although pressure exerted by neighbours is often measured as the rate of between-group encounters^81,82^, low rates might either represent low outsider pressure or indicate avoidance by rivals. Accordingly, using a plurality of metrics will be necessary to capture variation in outsider conflict within and between species; for any given comparison, the same metrics will be needed for all species being considered. Those metrics could be used as independent factors in analyses, potentially comparing between different threat types, or could be combined to form an index of overall threat. Recent work on between-group interactions in chimpanzees, for instance, has developed a single ‘neighbour pressure index’, integrating estimates of between-group interaction frequency, territorial position (with intrusions to the territory core being viewed as more threatening than those on the periphery) and disputed resource value (as indicated by usage by the resident group)^18^. This index was then coupled with a group’s competitive ability and within-group competition to consider influences on reproductive output^18^. It is this integrated approach, considering both interactions within groups and with outsiders, that we are advocating with respect to cognition.

Careful thought is also needed about the categorisation of outsiders and when these outsider metrics should be measured. In the majority of cases, most obviously in those species that defend all-purpose territories, the classification of outsiders is clear: it is those individuals not part of the territorial unit (be that the individual, pair or group). In some species, individuals breed in pairs, defending a nest site or breeding territory together, but feed with others in temporary groups; here, outsiders in the breeding context may be groupmates while foraging, so we would advocate measurement of threat metrics with respect to just the former. Likewise, there are examples of group-living species that require further consideration as to what constitutes an outsider (e.g., fission–fusion societies, where group composition shifts dynamically as groups divide into sub-groups and later reassemble). In these cases, it might be valuable to quantify outsider threats at multiple levels of social organisation, as is already often the case with neighbours vs. strangers in territorial species.

### Complementary approaches

With respect to intraspecific investigations of Napoleonic intelligence, we suggest three priority areas. First, we advocate a focus on the causes of individual variation in cognitive traits in relation to outsider interactions. This can be achieved by testing cognitive performance on psychometric tasks^7,83^ or using neuroanatomical measures, such as brain size^84^, size of brain regions^85^ or neuron density^86^. In addition, longitudinal studies, quantifying cognition at regular time steps over the course of an individual’s life^87^, may help to identify an effect of outsider threat on cognitive development. Quantifying individual cognitive performance in both domain-general cognitive tasks (e.g., associative learning^87^) and socio-cognitive tasks (e.g., social competence^88^) will be important in determining whether the cognitive consequences of outsider interactions are domain specific or if a ‘general intelligence factor’ exists. Second, investigation into the consequences of individual variation in cognition will help to determine whether outsider threats can have evolutionary implications. This can be achieved by examining the relationship between cognition and measures of fitness (such as reproductive success^87^). Third, comparisons of sub-populations experiencing different selection pressures offer a window into the factors governing cognitive variation^89^. As with predation risk^85^, natural variation in outsider threat levels provides one option and it may also be possible to manipulate experimentally the level of threat. Recent studies examining the short-term consequences of outgroup conflict, in both laboratory and field settings, have demonstrated the feasibility of simulating outsider threats using call playbacks, faecal presentations and controlled intrusions^80,90,91^.

Interspecific approaches, using phylogenetic comparisons at varying scales, can complement intraspecific studies by revealing long-term evolutionary trends in cognitive evolution^2,3^. Using phylogenetic regressions, measures of brain size (relative or total brain size, or size of certain brain regions) may be modelled as a function of outsider threat as determined from the metrics outlined in Fig. 3. The use of neuroanatomical measures as cognitive indicators is a controversial issue^92^ (e.g., links between brain size and cognitive performance have been identified in some cases^74,84,93^, but other researchers have argued that is unclear how they translate to differences in cognition^92^). However, these proxies are the most viable option currently available for large-scale comparisons (e.g., across birds^3^ or primates^2^). In addition, small-scale phylogenetic comparisons on clades of closely related species could use cognitive performance quantified in all the relevant species using identical psychometric methods and tests. Quantifying cognitive performance within a radiation would allow comparative analyses both to use direct measures of cognition and to mitigate the confounding effects that arise in making interspecific comparisons across disparate taxa. A combination of intra- and interspecific techniques would allow a powerful test of the central predictions about Napoleonic intelligence specifically and the expanded SIH more generally.

## Conclusion

For over 50 years, sociality has been hypothesised as an evolutionary driver of animal cognition^5,6^. However, the focus has been predominantly on interactions occurring within groups. Although this focus has advanced our understanding of cognition in diverse taxa^94^, the complexity of social interactions with conspecific outsiders (and their often surprising consequences for social evolution) is increasingly being recognised^65,70,95–97^. Conflict with conspecific outsiders is prevalent across the social spectrum (from individuals to pairs, to groups) and represents a powerful selective force; adaptations have arisen that minimise the risks and allow exploitation of the opportunities arising from these outsider interactions. We argue that a complementary focus of the SIH should therefore be those pressures arising from conspecific outsider interactions: considering both major axes of sociality will likely improve our understanding of social intelligence and cognitive evolution.

## Data Availability

No new data were used in the preparation of this manuscript.
