# Author Correction: Interactions with conspecific outsiders as drivers of cognitive evolution

**DOI:** 10.1038/s41467-020-20090-7

**Published:** 2020-11-24

**Authors:** Benjamin J. Ashton, Patrick Kennedy, Andrew N. Radford

**Affiliations:** 1 School of Biological Sciences, University of Bristol, 24 Tyndall Avenue, Bristol, BS8 1TQ UK; 2 Department of Biological Sciences, Macquarie University, Sydney, NSW 2109 Australia

**Keywords:** Behavioural ecology, Social evolution, Social behaviour, Animal behaviour

Correction to: *Nature Communications* 10.1038/s41467-020-18780-3, published online 6 October 2020.

The original version of this Article contained an error in Fig. 3 legend, which incorrectly read ‘Chimpanzee photograph: L. Samuni’. The correct version states ‘A. Sandel’ in place of ‘L. Samuni’. This has been corrected in both the PDF and HTML versions of the Article.

